# Gender disparities in all-cause mortality among individuals with early-onset cardiovascular diseases

**DOI:** 10.1186/s12889-024-18908-w

**Published:** 2024-05-30

**Authors:** Jing Yang, Shouling Wu, Yang Liu, Jinguo Jiang, Shuohua Chen, Boheng Zhang, Wei Li, Qi Zhang

**Affiliations:** 1 Department of Cardiology, Tangshan Gongren Hospital, No. 27, Wenhua Road, Lubei District, Tangshan, 063000 Hebei Province People’s Republic of China; 2 Department of Cardiology, Kailuan General Hospital, Tangshan, 063000 Hebei China; 3 Department of Epidemiology and Biostatistics, School of Public Health, Key Laboratory of Epidemiology of Major Diseases (Peking University), Ministry of Education, Peking University, 38# Xueyuan Road, Haidian District, Beijing, 100191 China; 4 Department of Clinical Epidemiology, Shengjing Hospital of China Medical University, Shenyang, Liaoning China; 5 Graduate School, Hebei Medical University, Shijiazhuang, China

**Keywords:** Kailuan study, Early-onset CVD, Disparity, Mortality

## Abstract

**Background and objective:**

Gender disparities in mortality among individuals with early-onset cardiovascular disease (CVD) remain uncertain. This study aimed to investigate gender differences in all-cause mortality and identify influencing factors.

**Methods:**

Data extracted from the Kailuan Study, a prospective cohort study initiated in 2006, were analyzed. A total of 2,829 participants with early-onset CVD were included. Cox proportional hazard models were used to assess hazard ratios (HR) and 95% confidence intervals (CI) for gender disparities in all-cause mortality, adjusting for various factors.

**Results:**

Males experienced a median follow-up duration of 7.54 years with 276 recorded deaths, and females had a median follow-up of 6.45 years with 105 recorded deaths. Gender disparities in all-cause mortality were observed, with men experiencing a higher all-cause mortality risk compared to women (HR: 1.42, 95% CI: 1.04, 1.92) in the fully adjusted model. Both in men and women with early-onset CVD, elevated hs-CRP levels and an eGFR < 60 mL/min/1.73m^2^ notably escalated the risk of all-cause mortality. Furthermore, the utilization of antiplatelet agents and successful blood glucose control might mitigate the risk of all-cause mortality. Smoking and eGFR decline modified the association between gender and all-cause death, women were more vulnerable to tobacco consumption and kidney misfunctioning than men (*P*-interaction = 0.019).

**Conclusion:**

The study highlights gender disparities in all-cause mortality among individuals with early-onset CVD, with men experiencing a higher risk of mortality compared to women. Addressing these disparities is important for improving outcomes in this population. Further research is needed to develop sex-specific interventions and strategies to reduce gender-related mortality disparities in early-onset CVD.

**Supplementary Information:**

The online version contains supplementary material available at 10.1186/s12889-024-18908-w.

## Introduction

According to the Global Burden of Disease Study in 2019, cardiovascular diseases (CVDs) account for over 30% of total deaths worldwide. Although the proportion of premature CVD deaths has declined among premature deaths, it remains as high as 40– [1–4]. A similar mode of death cause, CVD contributed to over 40% of total mortality, observed in China [5]. While the premature mortality rate related to CVD has shown a decreasing trend, the proportion of premature CVD deaths among premature deaths is still around 30% [6], [7].

Previous studies have found potential gender differences in overall mortality following premature CVD, but the results regarding gender disparities are inconclusive. A meta-analysis investigating gender differences in mortality rates after ST-segment elevation myocardial infarction (MI) showed higher 1-year mortality rates in women compared to men [8]. Young women (< 65 years) had a 1.843-fold higher risk of overall mortality following acute MI compared to young men (< 55 years) [9]. However, other studies have indicated lower 1-year mortality rates in women compared to men after acute ST-segment elevation MI [10], though long-term mortality rates beyond 1 year did not show significant gender differences [8]. Women also face a higher overall mortality risk after stroke compared to men^11,12^. However, gender may not be an independent risk factor for overall mortality in ischemic stroke (IS) populations [13]. Inconsistencies in gender differences in overall mortality following CVD may be attributed to the lack of consideration of age^14,15^, as women not only experience CVD at a later age than men but also have a longer average lifespan [16].

To clarify whether there are gender differences in overall mortality following new-onset premature CVD events, we conducted an observational study based on the Kailuan cohort to investigate the presence of gender disparities in all-cause mortality among individuals with early onset CVD and identify factors influencing these differences.

## Methods

### Participants and study design

The Kailuan Study is an ongoing prospective cohort study initiated in 2006. The detailed study design of the Kailuan Study has been described elsewhere [17]. The study conducts biennial follow-up visits and annually confirms cardiovascular events, including stroke, intracerebral hemorrhage, acute MI, as well as all-cause mortality. These conditions enable us to analyze factors influencing mortality following early-onset cardiovascular events in different genders. In this study, we included premature cardiovascular patients from the Kailuan Study. The period of observation from the diagnosis time of premature CVD until either all-cause mortality or December 31, 2020.

During the period from June 2006 to December 31, 2020, a total of 3,087 individuals who developed an early-onset CVD event (< 55 years for men^14 15^, < 65 years for women [18–20]) were included. And then excluding 258 patients with incomplete hospitalization data after the initial early-onset CVD event until December 31, 2020, a total of 2,829 participants were included for final analysis.

The study adhered to the guidelines of the Declaration of Helsinki and received ethical approval from the Ethics Review Committee of Kailuan General Hospital (No.2006-05). Signed Informed consent was obtained from all study participants.

### Data collection

Trained medical workers completed the standardized questionnaire through face-to-face interviews during participants’ health check-ups. The questionnaire meticulously recorded demographic details (date of birth, gender, and age), lifestyle (alcohol intake and tobacco consumption), personal medical history (hypertension, diabetes, MI, atrial fibrillation (AF), heart failure (HF), stroke, and malignant tumor), as well as medication history. Anthropometric assessments included height (centimeter), weight (kilograms), systolic blood pressure (mmHg), and diastolic blood pressure (mmHg). All participants provided a fasting venous blood sample of 5 mL from the antecubital vein into an ethylenediaminetetraacetic acid tube in the morning. Within 4 h, the levels of low-density lipoprotein cholesterol (LDL-C, mmol/L), creatinine (Cr, µmol/L), fasting blood glucose (FBG, mmol/L), and high-sensitivity C-reactive protein (hs-CRP, mg/L) were measured using an auto-analyzer (Hitachi 747; Hitachi, Tokyo, Japan). The hs-CRP levels were determined using an immunoturbidimetric assay. The procedures were strictly conducted by laboratory technicians following the instructions. The estimated glomerular filtration rate (eGFR) was calculated utilizing the Chronic Kidney Disease Epidemiology Collaboration Eq. 21, incorporating creatinine, sex, and age.

### Assessment of early-onset CVD

Men were considered to have early-onset CVD if the onset occurred before the age of 55^14^,^15^ while for women, the age criterion was set before 65^18^–^20^. Early-onset CVD events comprised AF, HF, acute MI, IS, and hemorrhagic stroke (HS). The disease diagnoses were determined based on the International Classification of Diseases, 10th Revision (ICD-10) codes. The codes for AF (ICD-10-CM): I48, HF (ICD-10-CM): I50.9, acute MI (ICD-10-CM): I21, IS (ICD-10-CM): I63, and HS (ICD-10-CM): I61. Information regarding these events was obtained from the inpatient medical records, inpatient medical record archives, and the medical insurance system. In the Kailuan study, all participants were enrolled through the Municipal Social Insurance Institution and Hospital Discharge Register as employees of the Kailuan Group. Monitoring for early-onset CVD was conducted by regularly updating these electronic records. Additionally, medical staff reviewed discharge lists from 11 hospitals and queried participants during biennial follow-up interviews using standardized questionnaires to identify suspected cases of early-onset CVD. Detailed diagnostic procedures have been elucidated in prior publications from the Kailuan study [22–25].

### Assessment of outcome

The primary outcome variable was all-cause mortality. Information on deaths during the follow-up period was obtained from the Kailuan social insurance system, including data on the time of death and survival status. All employees of the Kailuan Group are covered by the social insurance system, providing medical insurance as a fundamental welfare benefit. Unless formal resignation procedures are completed, Kailuan employees remain enrolled in this welfare program.

### Definition of covariates

Hypertension: According to the 2018 “Chinese Guidelines for the Prevention and Treatment of Hypertension,” the criteria for hypertension are as follows: in the absence of antihypertensive medication, blood pressure measurements taken on different days in the clinic show systolic blood pressure (SBP) ≥ 140 mmHg and/or diastolic blood pressure (DBP) ≥ 90 mmHg. Patients with a history of hypertension who are currently using antihypertensive medication and have blood pressure below 140/90 mmHg should still be diagnosed with hypertension [26]. Blood pressure target levels for patients with early-onset CVD [26, 27]: For individuals with IS, HS, subarachnoid hemorrhage, or MI, the blood pressure target is < 140/90 mmHg. For patients with heart failure or those with CVD combined with diabetes, the blood pressure target is < 130/80 mmHg. For individuals with AF, the blood pressure target range is 120–130/<80 mmHg. Diabetes: A diagnosis of diabetes is made if the fasting blood glucose level is ≥ 7.0 mmol/L or if the fasting blood glucose level is < 7.0 mmol/L but there is a documented history of diabetes or current use of antidiabetic medication [28]. High LDL-C Levels are defined as LDL-C levels ≥ 4.14 mmol/L (160 mg/dL) [29]. An eGFR of less than 60 mL/min/1.73 m² is defined as a decline in eGFR. Smoking is defined as the average consumption of at least one cigarette per day for more than one year within the past year. Alcohol consumption: Drinking is defined as an average consumption of at least 100 mL per day of spirits (with an alcohol content > 50%) within the past year.

Obesity (BMI, kg/m²)^30^: Obesity is defined as a BMI (Body Mass Index) of 30.0 kg/m² or higher. All variables were derived from the most recent physical examination or hospital admission at the time of early-onset CVD diagnosis.

### Statistical analysis

Normally distributed continuous data are presented as mean ± standard deviation, with intergroup comparisons conducted using independent sample t-tests. Skewed distributed continuous data are expressed as median (P25, P75)], with intergroup comparisons analyzed through non-parametric tests. Categorized variables are presented as n (percentage), and differences are assessed using the χ² test. The incidence density of all-cause mortality after early-onset CVD per 1000 person-years is calculated by dividing the number of events by the total follow-up person-years.

Cox regression models with age as the time scale were used to evaluate HR and 95% CI for gender disparities in all-cause mortality following incident early-onset CVD and additionally explore influencing factors. Cox models met the proportional hazard assumption before establishing. Model 1 adjusted for age (as the time scale); Model 2 adjusted for higher education, alcohol consumption, and smoking; Model 3 adjusted for obesity, hypertension, diabetes, high LDL-C levels, decreased eGFR, and elevated hs-CRP; Model 4 adjusted for antihypertensive, antidiabetic, lipid-lowering medications, antiplatelet agents, achieving blood pressure, glycemic, and lipid targets. Additionally, joint effects of gender and baseline characteristics were assessed. Interaction terms were further incorporated into the Cox model.

To validate the robustness of the primary results, we conducted further sensitivity analyses. Given the lower use of antiplatelet agents in the HS population, we excluded this subgroup. Additionally, due to the inability to assess the baseline severity of CVD in the study population, we conducted separate observations for individuals surviving beyond 30 days and those surviving up to 1 year after early-onset CVD events. Furthermore, the criterion for glycemic target was further adjusted to fasting blood glucose < 8.6 mmol/L because of the lack of glycated hemoglobin data.

Statistical analyses were performed using SAS 9.4 software. Two-tailed tests were employed, with statistical significance set at *P* < 0.05.

## Results

### Basic characteristics of the study population

A total of 2,829 early-onset CVD cases meeting the inclusion criteria with complete data were enrolled in this study, and 1,984 patients were males (70.13%). The average age for males was 49.48 ± 5.35 years, while for females, it was 57.05 ± 5.71 years. Overall, males exhibited higher DBP and eGFR than females; additionally, men exhibited lower levels of blood glucose, low-density lipoprotein cholesterol, and hs-CRP compared to females. A higher proportion of males had attained higher education, engaged in smoking and alcohol consumption, had hypertension, used lipid-lowering drugs, and were prescribed antiplatelet agents in comparison to females. In contrast, the proportion of diabetes and antidiabetic medications was lower in males than in females (Table 1).

**Table 1:** Baseline characteristics of early-onset CVD participants according to gender

	Total(*N* = 2829)	Male (*N* = 1984)	Female (*N* = 845)	*P* value
Age, years	51.74 ± 6.47	49.48 ± 5.35	57.05 ± 5.71	< 0.001
Higher education level, n (%)	168(5.94)	132(6.65)	36(4.26)	0.014
Smoking, n (%)	1266(44.75)	1224(61.69)	42(4.97)	< 0.001
Alcohol drinking, n (%)	1020(36.06)	978(49.29)	42(4.97)	< 0.001
Hypertension, n (%)	2303(81.41)	1641(82.71)	662(78.34)	0.006
Diabetes, n (%)	1011(35.74)	657(33.11)	354(41.89)	< 0.001
BMI, kg/m^2^	26.20 ± 3.69	26.18 ± 3.62	26.24 ± 3.84	0.700
SBP, mmHg	139.38 ± 22.40	138.88 ± 21.83	140.56 ± 23.66	0.078
DBP, mmHg	87.10 ± 12.59	88.30 ± 12.82	84.26 ± 11.56	< 0.001
FBG, mmol/L	6.50 ± 2.85	6.41 ± 2.74	6.68 ± 3.10	0.029
LDL-C, mmol/L	2.60 ± 0.95	2.58 ± 0.93	2.67 ± 1.01	0.021
eGFR, mL/min/1.73m^2^	90.46 ± 23.39	92.58 ± 22.41	85.48 ± 24.86	< 0.001
hs-CRP, mg/L	1.80(0.70, 4.65)	1.70(0.62, 4.34)	2.20(0.80, 5.00)	< 0.001
Antihypertensive drugs, n (%)	1900(67.16)	1358(68.45)	542(64.14)	0.026
*Hypoglycemic drugs, n (%)	672(66.47)	406(61.80)	266(75.14)	< 0.001
lipid-lowering drugs, n (%)	1283(45.35)	929(46.82)	354(41.89)	0.016
Antiplatelet drugs, n (%)	1166(41.22)	859(43.30)	307(36.33)	< 0.001
Blood pressure up to standard, n (%)	1253(44.29)	868(43.75)	385(45.56)	0.375
Blood glucose up to standard, n (%)	2152(76.07)	1527(76.97)	625(73.96)	0.087
Lipid up to standard, n (%)	565(19.97)	403(20.31)	162(19.17)	0.487

*, Early-onset CVD participants with diabetes

### Association between gender and all-cause mortality in the early-onset CVD population

After early-onset cardiovascular disease (CVD) events, males exhibited a median follow-up duration of 7.54 years (IQR: 3.90, 11.12) with 276 recorded deaths, whereas females had a median follow-up of 6.45 years (IQR: 3.34, 10.16) with 105 recorded deaths. Adjusting for covariates in model 2, men displayed a significantly increased risk of all-cause mortality compared to females (HR: 1.47, 95% CI: 1.08, 1.99). Similar results were also observed in model 3 (HR: 1.42, 95% CI: 1.04, 1.92) (Table 2). Notably, individuals with HS exhibited a lower proportion of antiplatelet agents use, and upon their exclusion, men with early-onset CVD continued to manifest a higher risk of all-cause mortality compared to females (HR: 1.49, 95% CI: 1.07, 2.08). Due to the absence of glycosylated hemoglobin (HbA1c) data, a value of 7% for HbA1c equated to a venous blood glucose level of 8.6mmol/L^28^, Con sequently, the covariate criterion for blood glucose control was adjusted to a fasting blood glucose level < 8.6 mmol/L. Results consistently pointed to a heightened risk of all-cause mortality in males with early-onset CVD compared to females (HR: 1.49, 95% CI: 1.09, 2.04). In the early-onset CVD population, both those surviving beyond 30 days and those surviving beyond 1 year displayed a higher risk of all-cause mortality in males compared to females (HR: 1.80, 95% CI: 1.29, 2.53; HR: 1.42, 95% CI: 1.04, 1.92) (Supplemental Table **S1**).

**Table 2:** Hazard ratios and 95% confidence intervals of gender-specific all-cause death among early-onset CVD participants

	Death/*N*	Incidence rate ( per 1000 person years)	HR(95% CI)		
			Model 1	Model 2	Model 3
Female	105/845	18.20	1.00(reference)	1.00(reference)	1.00(reference)
Male	276/1984	18.37	1.23(0.72, 2.09)	**1.47(1.08, 1.99)**	**1.42(1.04, 1.92)**

Model 1 was adjusted for age (as time scale);

Model 2: model 1 + higher education level (illiterate, primary, middle school, high school, and above high school), alcohol drinking(never or former drink, current drink), smoking (never or former smoke, current smoke), obese (BMI ≥ 30.0 kg/m^2^), hypertension (yes or no), diabetes (yes or no), high LDL-C (LDL-C ≥ 4.14mmol/L), eGFR decline (eGFR < 60mL/min·1.73m^2^), elevated hs-CRP (hs-CRP > 3 mg/L);

Model 3: model 2 + antihypertensive drugs (yes or no), hypoglycemic drugs (yes or no), lipid-lowering drugs (yes or no), antiplatelet drugs (yes or no), blood pressure up to standard, blood glucose up to standard, lipid up to standard

### Factors influencing all-cause mortality in early-onset CVD populations of different genders

**Fig. 1:**
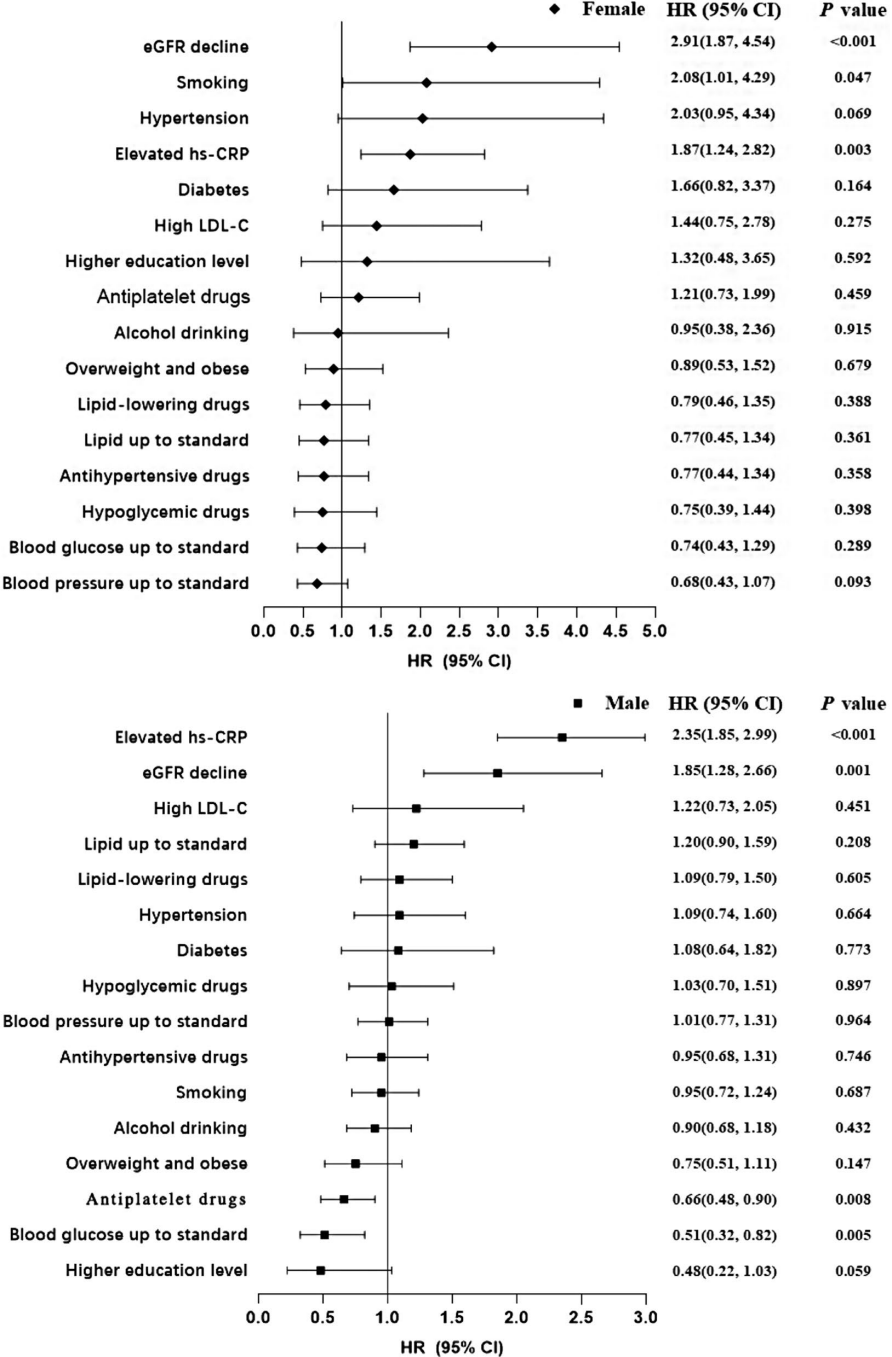
Risk factors of gender-specific all-cause death among early-onset CVD participants. Model was adjusted for age(as time scale), higher education level, alcohol drinking (never or former drink, current drink), smoking (never or former smoke, current smoke), obese (BMI ≥ 30.0 kg/m^2^),, hypertension (yes or no), diabetes (yes or no), high LDL-C (LDL-C ≥ 4.14mmol/L), eGFR decline (eGFR < 60mL/min·1.73m^2^), elevated hs-CRP (hs-CRP > 3 mg/L), antihypertensive drugs (yes or no), hypoglycemic drugs (yes or no), lipid-lowering drugs (yes or no), and antiplatelet drugs (yes or no), blood pressure up to standard, blood glucose up to standard, lipid up to standard

In males with early-onset CVD, elevated hs-CRP levels and an eGFR < 60 mL/min/1.73m^2^ notably escalated the risk of all-cause mortality (HR: 2.35, 95% CI: 1.85, 2.99; HR: 1.85, 95% CI: 1.28, 2.66). Furthermore, the utilization of antiplatelet agents and successful blood glucose control significantly mitigated the risk of all-cause mortality (HR: 0.66, 95% CI: 0.48, 0.90; HR: 0.51, 95% CI: 0.32, 0.82). Among females with early-onset CVD, an eGFR < 60 mL/min/1.73m^2^, smoking, and elevated hs-CRP levels significantly heightened the risk of all-cause mortality (HR: 2.91, 95% CI: 1.87, 4.54; HR: 2.08, 95% CI: 1.01, 4.29; HR: 1.87, 95% CI: 1.24, 2.82) (Table 3; Fig. 1).

**Table 3:** Risk factors of gender-specific all-cause death among early-onset CVD participants

	Female	Male
Higher education level	1.32(0.48, 3.65)	0.48(0.22, 1.03)
Smoking	**2.08(1.01, 4.29)**	0.95(0.72, 1.24)
Alcohol drinking	0.95(0.38, 2.36)	0.90(0.68, 1.18)
Overweight and obese	0.89(0.53, 1.52)	0.75(0.51, 1.11)
Hypertension	2.03(0.95, 4.34)	1.09(0.74, 1.60)
Diabetes	1.66(0.82, 3.37)	1.08(0.64, 1.82)
High LDL-C	1.44(0.75, 2.78)	1.22(0.73, 2.05)
eGFR decline	**2.91(1.87, 4.54)**	**1.85(1.28, 2.66)**
Elevated hs-CRP	**1.87(1.24, 2.82)**	**2.35(1.85, 2.99)**
Antihypertensive drugs	0.77(0.44, 1.34)	0.95(0.68, 1.31)
Hypoglycemic drugs	0.75(0.39, 1.44)	1.03(0.70, 1.51)
Lipid-lowering drugs	0.79(0.46, 1.35)	1.09(0.79, 1.50)
Antiplatelet drugs	1.21(0.73, 1.99)	**0.66(0.48, 0.90)**
Blood pressure up to standard	0.68(0.43, 1.07)	1.01(0.77, 1.31)
Blood glucose up to standard	0.74(0.43, 1.29)	**0.51(0.32, 0.82)**
Lipid up to standard	0.77(0.45, 1.34)	1.20(0.90, 1.59)

Model was adjusted for age(as time scale), higher education level, alcohol drinking (never or former drink, current drink), smoking (never or former smoke, current smoke), obese (BMI ≥ 30.0 kg/m^2^), hypertension (yes or no), diabetes (yes or no), high LDL-C (LDL-C ≥ 4.14mmol/L), eGFR decline (eGFR < 60mL/min·1.73m^2^), elevated hs-CRP (hs-CRP > 3 mg/L), antihypertensive drugs (yes or no), hypoglycemic drugs (yes or no), lipid-lowering drugs (yes or no), and antiplatelet drugs (yes or no), blood pressure up to standard, blood glucose up to standard, lipid up to standard

### Stratified and interaction analysis of the baseline characteristics of the early-onset CVD population

An interaction between gender and smoking (*P*-interaction = 0.019), as well as between eGFR decline (*P*-interaction = 0.032), was observed. In comparison to non-smoking females, both smoking women (HR: 2.12, 95% CI: 1.14, 3.95) and men (HR: 1.53, 95% CI: 1.09, 2.15) showed a substantial increase in the risk of all-cause mortality. Similarly, compared to females without eGFR decline, both females (HR: 3.38, 95% CI: 2.23, 5.11) and males (HR: 3.05, 95% CI: 1.96, 4.74) with an eGFR < 60mL/min/1.73m^2^ exhibited a significantly higher risk of all-cause mortality (Table 4).

**Table 4:** Joint effect of gender and baseline characteristics on all-cause death among early-onset CVD participants

		Cases	Female	Male	*P* value for interaction
Higher education level	No	2661	1.00(reference)	1.45(1.07, 1.97)	0.156
	Yes	168	1.20(0.44, 3.27)	0.70(0.31, 1.56)	
Smoking	No	1563	**1.00(reference)**	**1.59(1.15, 2.19)**	**0.019**
	Yes	1266	**2.12(1.14, 3.95)**	**1.53(1.09, 2.15)**	
Alcohol drinking	No	1809	1.00(reference)	1.47(1.07, 2.01)	0.340
	Yes	1020	1.31(0.60, 2.89)	1.30(0.89, 1.89)	
obese	No	1714	1.00(reference)	1.46(1.06, 2.00)	0.542
	Yes	1115	0.89(0.53, 1.49)	1.06(0.67, 1.70)	
Hypertension	No	526	1.00(reference)	**2.61(1.28, 5.30)**	**0.055**
	Yes	2303	**2.25(1.14, 4.45)**	**2.88(1.45, 5.72)**	
Diabetes	No	1818	1.00(reference)	1.48(1.01, 2.19)	0.697
	Yes	1011	1.35(0.80, 2.27)	1.82(1.07, 3.10)	
High LDL-C	No	2666	1.00(reference)	1.46(1.07, 1.99)	0.395
	Yes	163	1.59(0.84, 2.99)	1.63(0.91, 2.91)	
eGFR decline	No	2586	**1.00(reference)**	**1.65(1.18, 2.32)**	**0.032**
	Yes	243	**3.38(2.23, 5.11)**	**3.05(1.96, 4.74)**	
elevated hs-CRP	No	1006	1.00(reference)	1.28(0.86, 1.91)	0.452
	Yes	1823	2.01(1.35, 2.97)	3.06(2.07, 4.54)	
Antihypertensive drugs	Yes	1900	1.00(reference)	1.40(0.99, 1.98)	0.878
	No	929	1.17(0.73, 1.86)	1.70(1.12, 2.57)	
*Hypoglycemic drugs	Yes	672	1.00(reference)	1.70(1.06, 2.73)	0.487
	No	339	1.12(0.60, 2.09)	1.49(0.86, 2.57)	
lipid-lowering drugs	Yes	1283	1.00(reference)	1.36(0.91, 2.02)	0.754
	No	1546	0.91(0.59, 1.39)	1.32(0.87, 2.01)	
Antiplatelet drugs	Yes	1448	1.00(reference)	1.09(0.72, 1.67)	**0.096**
	No	1381	0.99(0.65, 1.52)	**1.61(1.04, 2.50)**	
Blood pressure up to standard	Yes	1694	1.00(reference)	1.82(1.16, 2.86)	0.124
	No	1135	1.47(0.96, 2.26)	1.83(1.19, 2.81)	
Blood glucose up to standard	Yes	2152	1.00(reference)	1.39(0.98, 1.97)	0.817
	No	677	1.61(1.01, 2.57)	2.36(1.51, 3.69)	
Lipid up to standard	Yes	245	1.00(reference)	**2.14(1.19, 3.82)**	**0.095**
	No	2584	1.35(0.80, 2.30)	**1.74(1.00, 3.02)**	

Early-onset CVD participants with diabetes.

Model was adjusted for age (as time scale), higher education level (illiterate, primary, middle school, high school, and above high school), alcohol drinking(never or former drink, current drink), smoking (never or former smoke, current smoke), obese (BMI ≥ 30.0 kg/m^2^), hypertension (yes or no), diabetes (yes or no), high LDL-C (LDL-C ≥ 4.14mmol/L), eGFR decline (eGFR < 60mL/min·1.73m^2^), elevated hs-CRP (hs-CRP > 3 mg/L), antihypertensive drugs (yes or no), hypoglycemic drugs (yes or no), lipid-lowering drugs (yes or no), and antiplatelet drugs (yes or no), blood pressure up to standard, blood glucose up to standard, lipid up to standard, except for stratification variables.

## Discussion

Our main finding indicates a higher risk of all-cause mortality in males than females within the early-onset CVD population. Factors leading to all-cause mortality differ between different gender groups. Smoking and eGFR decline can increase the risk of all-cause mortality in females with early-onset CVD while achieving blood glucose control and using antiplatelet drugs can reduce the risk of all-cause mortality in males with early-onset CVD.

Previous findings on gender disparities in all-cause mortality among CVD patients have shown inconsistent results. Some studies indicated a lower mortality risk for women compared to men in patients with acute MI and stroke (HR: 0.76, 95% CI: 0.61, 0.95; HR: 0.83, 95% CI: 0.78, 0.98)^10^,^31^. However, studies from China and the United States found no gender differences in all-cause mortality among CVD patients (OR: 0.65, 95% CI: 0.38, 1.11; OR: 1.04, 95% CI: 0.84, 1.29)^13,32^. Conversely, a study from Korea even reported the opposite conclusion, identifying a higher mortality risk for young women than men in acute MI populations (HR: 1.84, 95% CI: 1.10, 3.01) [9]. In contrast to previous research, our observation pertains to early-onset CVD patients, revealing for the first time that women have a lower risk of mortality than men within the early-onset CVD population (HR: 0.71, 95% CI: 0.52, 0.96).

we found that different risk factors have varying degrees of impact on all-cause mortality in different gender groups with early-onset CVD. There exists an interaction between smoking, eGFR decline, and gender on the risk of all-cause mortality in early-onset CVD (*P*-interaction = 0.019; *P*-interaction = 0.032).

The effects of smoking and eGFR decline on increasing the risk of all-cause mortality in females with early-onset CVD are stronger than in males (HR: 2.12 versus 1.53; HR: 3.38 versus 3.05). Our findings align with previous research. Smoking cessation interventions in the United States and preventive guidelines suggest that the health impact of smoking is more significant in females compared to males [33], and women are more susceptible to tobacco toxicity [34]. An international meta-analysis revealed that as kidney function declines, the increase in all-cause mortality and cardiovascular mortality rates in females is faster than in males. Compared to those with better kidney function (eGFR top 95%), females with poorer kidney function (eGFR bottom 45%) had a significantly higher risk of all-cause mortality (HR: 1.32, 95% CI 1.08, 1.61) than males (HR: 1.22, 95% CI: 1.00, 1.48) (*P*-interaction < 0.01). Women often exhibit greater sensitivity to declining kidney function [35]. Our results expand upon previous evidence regarding the differential impact of smoking and low eGFR on adverse outcomes in different gender groups.

The 2021 ESC Clinical Practice Guidelines for Cardiovascular Disease Prevention indicated that aspirin reduces total mortality by 10% in atherosclerotic disease patients [36]. Similarly, our study found a significantly increased risk of mortality in early-onset CVD males who did not use antiplatelet drugs (HR: 1.61, 95% CI: 1.04, 2.50). Early use of antiplatelet drugs may yield greater health benefits. However, the impact of antiplatelet drugs on females was not substantial (HR: 0.99, 95% CI: 0.65, 1.52), potentially due to the smaller sample size of females in this study, resulting in insufficient statistical power to detect significant differences in the use of antiplatelet drugs in the female population. The mortality risk did not differ between early-onset CVD males using antiplatelet drugs and females using antiplatelet drugs (HR: 1.09, 95% CI: 0.72, 1.67). Thus, for both male and female patients with early-onset CVD, adherence to antiplatelet therapy following CVD prevention guidelines is recommended to achieve greater clinical benefits.

Through subgroup analyses of various risk factors, we found that gender differences result from the interaction of multiple factors, primarily reflecting differences in physiology and lifestyle between men and women. The diagnostic and treatment processes for early-onset CVD populations may require separate assessment systems to accurately evaluate the health status of these individuals. Our findings highlighting the higher mortality risk in males with early-onset CVD hold significant implications for public health and clinical practice. They provide crucial evidence for implementing gender-specific measures and assessment systems tailored to public health strategies and clinical treatments for individuals with early-onset CVD.

### Limitations

Our research was conducted within a large-scale cohort in the Kailuan community, supported by comprehensive health check-ups and national insurance. This approach facilitated the timely recording of the early health status of individuals developing CVD, allowing for a robust observation of the relationship between early-onset CVD and mortality outcomes. Moreover, our study spanned a lengthy follow-up period, from 2006 to 2020, involving a dynamic cohort that provided a substantial sample size of individuals with early-onset CVD for comprehensive research outcomes exploration. Despite numerous advantages, our study has limitations. First, while the homogeneity of the Kailuan cohort enhanced the robustness of our findings, it couldn’t account for population and ethnic diversity. Future validation of our outcomes might necessitate multi-center cohorts across diverse regions. Second, our study had a higher representation of male patients than females. However, our comprehensive analysis of gender differences in future mortality risk among early-onset CVD individuals confirmed consistent results, further solidifying the observed gender-based disparity in early-onset CVD. Lastly, although we accounted for demographic, lifestyle, metabolic, comorbidity, and medication factors in our study, there may still be unadjusted potential variables to consider.

## Conclusion

In the early-onset CVD population, the risk of mortality is higher in males than in females. Therefore, future health assessments and explorations of risk factors for early-onset CVD might need gender-stratified to achieve maximum cost-effectiveness. Simultaneously, this emphasizes the need for gender-specific evaluations in the future construction of health assessment systems and clinical guidelines for individuals with early-onset CVD.

### Electronic supplementary material

Below is the link to the electronic supplementary material.

Supplementary Material 1

## Data Availability

The original data analyzed in this research can be made available with permission of the corresponding author.

## Abbreviations

CVD: Cardiovascular Disease
HR: Hazard Ratios
CI: Confidence Intervals
eGFR: Estimated Glomerular Filtration Rate
CRP: C-reactive Protein
MI: Myocardial Infarction
AF: atrial fibrillation
HF: Heart Failure
IS: Ischemic
HS: Hemorrhagic Stroke
LDL-C: Low-density Lipoprotein Cholesterol
FBG: Fasting Blood Glucose
