# Supplementary material for: Gender disparities in all-cause mortality among individuals with early-onset cardiovascular diseases
Source: BMC Public Health. 2024 May 30;24:1450. doi: 10.1186/s12889-024-18908-w (PMC11140924; doi:10.1186/s12889-024-18908-w)
Supplement: Supplementary file 1 — Supplementary Material 1 [file 12889_2024_18908_MOESM1_ESM.docx]

| Supplemental Table S1. Sensitive analyses for hazard ratios and 95% confidence intervals of gender-specific all-cause death among early-onset CVD participants | | | | |
| --- | --- | --- | --- | --- |
|  | Sensitive analyses 1 | Sensitive analyses 2 | Sensitive analyses 3 | Sensitive analyses 4 |
| Female | 1.00(reference) | 1.00(reference) | 1.00(reference) | 1.00(reference) |
| Male | 1.49(1.07, 2.08) | 1.49(1.09, 2.04) | 1.80(1.29, 2.53) | 1.42(1.04, 1.92) |
| Footnotes: Model was adjusted for age(as time scale), higher education level, alcohol drinking (never or former drink, current drink), smoking (never or former smoke, current smoke), obese (BMI≥30.0kg/m2), hypertension (yes or no), diabetes (yes or no), high LDL-C (LDL-C≥4.14mmol/L), eGFR decline (eGFR<60mL/min·1.73m2), elevated hs-CRP (hs-CRP>3mg/L), antihypertensive drugs (yes or no), hypoglycemic drugs (yes or no), lipid-lowering drugs (yes or no), and antithrombotic drugs (yes or no), blood pressure up to standard, blood glucose up to standard, lipid up to standard. Sensitive analyses 1 was for the participants without hemorrhagic stroke. Sensitive analyses 2 was for the participants without death within 30 days. Sensitive analyses 3 was for the participants without death within 1 year. Sensitive analyses 4 changed the definition of blood glucose up to standard (Fbg<8.6mmol/L). | | | | |
